# Assessment of Telemedicine Perceptions, Usability, and Implementation Barriers Among Physicians in Kazakhstan Using the Telehealth Usability Questionnaire-Model for Assessment of Telemedicine-Kazakhstan Version (TUQ-MAST-KZ) Questionnaire: Pilot Cross-Sectional Survey Study

**DOI:** 10.2196/83667

**Published:** 2026-04-07

**Authors:** Kulzhamila Kenessova, Mariya Anartayeva, Kanatzhan Кеmelbekov, Aizat Seidakhmetova, Altynshash Kushkarova, Talgat Rysbekov, Aigul Sultangaziyeva

**Affiliations:** 1 JSC South Kazakhstan Medical Academy, Al-Farabi Square, 1, Shymkent, 160000, Kazakhstan, 7 87058528373; 2 Asfendiyarov Kazakh National Medical University, Almaty, Kazakhstan

**Keywords:** telemedicine, digital health, implementation barriers, TUQ-MAST-KZ, physicians’ perception, Kazakhstan, digital maturity

## Abstract

**Background:**

Health care professionals’ perceptions of telemedicine, its usability, and the presence of organizational barriers are important determinants of the successful implementation of digital solutions in health care. In Kazakhstan, the use of international assessment instruments requires contextual adaptation. The Telehealth Usability Questionnaire-Model for Assessment of Telemedicine-Kazakhstan version (TUQ-MAST-KZ) questionnaire was previously developed and psychometrically validated by integrating elements of the TUQ and MAST frameworks to assess perceptions of telemedicine within the national context.

**Objective:**

The aim of this study was to conduct the first pilot application of the TUQ-MAST-KZ questionnaire with physicians in Kazakhstan and perform an initial assessment of the organizational, technical, and educational aspects of telemedicine implementation.

**Methods:**

This cross-sectional study involved an anonymous online survey using the TUQ-MAST-KZ questionnaire, which covers perceptions of telemedicine, formats of use, platform usability, communication-related aspects, telemonitoring, organizational conditions, and implementation barriers. Responses from 156 physicians were analyzed. Stratified nonparametric comparisons were performed by sex, age group, work experience (years), and workplace, adjusted for multiple comparisons.

**Results:**

The most used telemedicine formats were telephone consultations (78/156, 50%), video consultations (69/156, 44.2%), chats and messaging applications (57/156, 36.5%), and mobile apps (48/156, 30.8%). The Kazakhstan National Telemedicine Network was used by 14.7% (23/156). Wearable devices were used by 5.8% (9/156). Telemedicine technologies incorporating artificial intelligence elements were used regularly by 13.5% (21/156) and occasionally by 32.1% (50/156) and not used by 50.6% (79/156). Positive ratings were as follows: 48.7% (76/156) regarding the simplicity and intuitiveness of telemedicine platforms; 56.4% (88/156) regarding the timeliness of patient condition monitoring; 51.9% (81/156) regarding the effectiveness of telemedicine for the management of patients with chronic diseases. The potential usefulness of telemonitoring for earlier detection of deterioration of a patient’s condition was rated as fairly or very high by 48.7% (76/156); 41% (64/156) rated it as moderate. Only 35.9% (56/156) positively rated the connection’s reliability and stability. Regarding the accuracy of wearable device data transmission, 57.1% (89/156) responded neutrally, potentially indicating ambiguity in perception, limited personal experience, or difficulty evaluating this aspect. Readiness to recommend telemonitoring at the national level was more often rated as moderate, high, or very high (78/156, 50%; 42/156, 26.9%; 14/156, 9%, respectively).

**Conclusions:**

This pilot application of the TUQ-MAST-KZ questionnaire showed a generally moderately positive perception of telemedicine by physicians, who recognized its potential clinical and organizational value. However, we identified substantial technical and institutional barriers, including connection instability, concerns about the accuracy of data transmission, insufficient process formalization, and a need for additional training. These preliminary findings should be interpreted in light of the pilot study design; however, they may serve to inform future larger-scale research and the development of organizational measures related to physician training, protocol standardization, and infrastructure support for telemedicine implementation.

## Introduction

The implementation of telemedicine and digital health services has become a priority area for health care modernization worldwide, including in the Republic of Kazakhstan [1,2]. Despite ongoing infrastructure development and the implementation of national initiatives, the adoption of telemedicine technologies is often constrained by a combination of organizational and human factors, including health care professionals’ attitudes toward innovation, readiness for training, and perceptions of digital services [3-7]. Positive attitudes and physician engagement facilitate overcoming technical and regulatory barriers, whereas a lack of motivation and trust reduces the effectiveness of implementation [6,8].

The importance of these factors is also supported within specific clinical fields. For example, the review by Tully et al [9] showed that, in pediatric telemedicine, successful implementation depends on users’ perceptions of the technology, organizational readiness, and the presence of factors that facilitate the acceptance of digital forms of interaction.

Traditionally, the effectiveness of telemedicine has been evaluated in terms of clinical, organizational, and economic aspects [5,10,11]. However, experience with scaling digital solutions has highlighted the central role of the human factor, particularly end users’ perceptions and practical readiness [4,6,10]. To systematically examine these dimensions, validated assessment instruments and approaches are used, including the Telehealth Usability Questionnaire (TUQ) [12] and the Model for Assessment of Telemedicine (MAST) [11], as well as approaches based on patient-reported outcome measures and patient-reported experience measures [10,13], which make it possible to assess the usability of, satisfaction with, and organizational value of telemedicine services. In particular, the TUQ is focused on usability and user experience, whereas the MAST framework extends the assessment by incorporating clinical, economic, and organizational parameters [11,12]. In addition, the TUQ has been translated, cross-culturally adapted, and validated in other countries, including Danish and German versions, which supports its applicability across different linguistic and cultural contexts [14,15].

Given the national context of Kazakhstan, there was a need to adapt international methodologies to local conditions. The Telehealth Usability Questionnaire-Model for Assessment of Telemedicine-Kazakhstan version (TUQ-MAST-KZ) instrument developed and validated by our team combines the strengths of the TUQ and MAST frameworks, making it possible to simultaneously assess perceptions of telemedicine, usability, satisfaction, and barriers affecting its use in clinical practice [16]. This approach is particularly relevant for health care organizations in Kazakhstan, including institutions participating in the Kazakhstan National Telemedicine Network (KNTN), a state system of remote consultations between health care organizations at different levels as well as organizations providing remote medical services under current regulatory requirements [17].

The need for a context-adapted instrument in Kazakhstan is driven by several factors. First, telemedicine practice in the country is developing in the context of pronounced heterogeneity in digital infrastructure, differences between urban and rural health care organizations, and uneven organizational readiness of medical institutions to implement digital solutions. Second, the specific functioning of the KNTN creates a distinct institutional context for the use of telemedicine consultations. Third, international instruments developed in other health care systems do not always fully capture the barriers and conditions relevant to physicians in Kazakhstan, including issues related to local protocols, the distribution of responsibilities, technical stability, and training needs. Accordingly, the use of the adapted TUQ-MAST-KZ questionnaire appears necessary for a more accurate assessment of telemedicine perceptions, usability, and implementation barriers in the national context.

This paper presents the results of the first pilot application of the previously developed and psychometrically validated TUQ-MAST-KZ questionnaire with a pilot sample of physicians in Kazakhstan [16]. Unlike the previous publication, which focused on the development and validation of the instrument, this study focused on its practical application for assessing perceptions of telemedicine, its usability, and implementation barriers in the national context. This work should not be regarded as a final national assessment but rather as a preliminary empirical basis for subsequent larger-scale research on the readiness for the implementation of telemedicine solutions and for discussing approaches to their phased expansion in Kazakhstan.

The aim of the study was to carry out the first pilot application of the TUQ-MAST-KZ questionnaire with a pilot sample of physicians in Kazakhstan and to provide an initial assessment of the organizational, technical, and educational aspects of telemedicine implementation.

## Methods

### Study Design

The study used a cross-sectional design and was conducted as an anonymous online survey. Data were collected using a Google Forms electronic questionnaire between May 30, 2025, and June 9, 2025.

### Ethical Considerations

The study protocol was approved by the Local Ethics Committee of South Kazakhstan Medical Academy (Protocol Number 3 dated April 25, 2025). All procedures were conducted in accordance with the principles of the Declaration of Helsinki and the requirements of national legislation [18,19]. The ethics approval document is provided in Multimedia Appendix 1.

Participation in the study was voluntary, anonymous, and uncompensated. Before completing the questionnaire, participants were provided with brief information about the purpose of the study, its voluntary nature, data confidentiality, and the use of the results in aggregated form only. Electronic informed consent was considered obtained once participants voluntarily proceeded to complete the questionnaire and submitted it. No personally identifiable data were collected.

### Participant Recruitment

Participants were recruited using a convenience nonprobability sampling approach among physicians working in health care organizations in the Republic of Kazakhstan. A link to the anonymous electronic questionnaire hosted on Google Forms was distributed through the researcher’s personal professional contacts and professional WhatsApp groups. Participants were also invited, if they wished, to forward the link to colleagues, thereby further extending the sample reach through network-based dissemination. No formal list of potential participants was compiled in advance, no centralized contact registry was used, and no individualized invitations were sent. The invitation included brief information about the purpose of the study and the conditions of participation.

Because the questionnaire was distributed through professional networks and group communication channels, the exact number of physicians who actually received the invitation to participate could not be determined. Accordingly, a valid calculation of the response rate was not possible. This is considered a methodological limitation of the study and a potential source of selection bias related to participant self-selection, as physicians with a greater interest in digital technologies, telemedicine, or innovation in health care practice may have been more likely to participate. In addition, physicians who were less engaged in digital professional communication, were not members of relevant professional groups, or were less inclined to participate in online surveys may have been underrepresented in the sample. This may have biased the results toward greater awareness of telemedicine and more favorable perceptions of digital solutions. Therefore, the findings should be interpreted with caution, and their generalizability to the overall population of physicians in Kazakhstan is limited.

### Inclusion Criteria

Eligible participants were physicians working in public or private health care organizations in the Republic of Kazakhstan who represented different levels of health care delivery, were proficient in Kazakh or Russian, and were willing to participate voluntarily in the survey. Prior practical experience with telemedicine technologies was not required, as one of the aims of the study was to assess differences in awareness and perceptions of telemedicine among physicians with different levels of familiarity with this field.

### Study Instrument

Data were collected using the TUQ-MAST-KZ questionnaire [16], which was developed based on the TUQ [12] and MAST [11]. The questionnaire consists of 27 items grouped into 7 domains: (1) clinical effectiveness, (2) user perceptions and satisfaction, (3) organizational aspects of implementation, (4) economic efficiency and resource implications, (5) legal safeguards and compliance with standards, (6) social impact and effects on patients, and (7) ethical and regulatory aspects of use. For the national context, additional items (Q7-Q9) were included to assess familiarity with telemedicine, formats of use, and experience with artificial intelligence (AI) technologies.

The translation and cultural adaptation of the TUQ-MAST-KZ questionnaire were carried out in accordance with international recommendations for the translation, cultural adaptation, and validation of instruments [20-22]. The procedure included forward and backward translation, expert review, and cognitive testing. Content validity was confirmed by an expert panel (n=6) using the item-level content validity index and scale-level content validity index (divided by the average) [22], and pilot testing demonstrated high internal consistency (Cronbach *α*=0.93). This study focused on the pilot application of the instrument among physicians in Kazakhstan. For this pilot application, the analysis focused primarily on items related to familiarity with telemedicine, formats of use, usability, communication and telemonitoring, organizational conditions, and implementation barriers; the full structure of the instrument is provided in Multimedia Appendix 2.

### Statistical Analysis

Statistical analysis was performed using SPSS version 27 (IBM Corp) and Excel (Microsoft Corp). Frequencies, percentages, means, and standard deviations were calculated. Spearman correlation coefficients were used to assess the associations between the level of familiarity with telemedicine (Q7) and selected telemedicine-related indicators (Q10-Q15). Statistical significance was set at *P*<.05.

Additional comparisons of responses were conducted according to sex, age group, years of work experience, and workplace (level of urbanization). Because responses on the Likert scale were ordinal in nature, the Mann-Whitney *U* test was used to compare 2 independent groups, whereas the Kruskal-Wallis test was used for comparisons across 3 or more independent groups. To reduce the risk of false-positive findings arising from multiple statistical testing, the Benjamini-Hochberg correction was applied. When differences across multiple groups reached statistical significance, additional pairwise comparisons were performed using the Holm correction. Differences were considered statistically significant at *P*<.05.

## Results

### Participant Characteristics

A total of 156 physicians were included in the study. Women predominated among the participants, accounting for 97 (97/156, 62.2%) of the physicians, while men comprised 59 (59/156, 37.8%) physicians. The age group with the highest number of physicians was 23‐30 years (50/156, 32.1%), followed by 31‐40 years (40/156, 25.6%) and 51‐60 years (36/156, 23.1%). In terms of work experience, the most represented categories were 3‐5 years (63/156, 40.4%) and >20 years (52/156, 33.3%). Most respondents worked in outpatient clinics (70/156, 44.9%) and hospitals (43/156, 27.6%). According to level of urbanization, physicians from metropolitan areas predominated (87/156, 55.8%). Detailed sample characteristics are presented in Table 1.

**Table 1.** Sociodemographic and professional characteristics of the respondents (n=156).

Question number and indicator	Results, n (%)
Q1. Sex	
Male	59 (37.8)
Female	97 (62.2)
Q2. Age (years)	
23‐30	50 (32.1)
31‐40	40 (25.6)
41‐50	24 (15.4)
51‐60	36 (23.1)
>60	6 (3.8)
Q3. Type of institution	
Outpatient clinic	70 (44.9)
Hospital	43 (27.6)
Medical center	25 (16)
Health administration	4 (2.6)
Dispensary	1 (0.6)
Medical university	8 (5.1)
Other	5 (3.2)
Q4. Work experience (years)	
3‐5	63 (40.4)
6‐10	16 (10.3)
11‐15	12 (7.7)
16‐20	13 (8.3)
>20	52 (33.3)
Q5. Place of work	
Rural area	25 (16)
Small town	15 (9.6)
Large city/urbanized area	29 (18.6)
Metropolitan city	87 (55.8)

### Distribution of Respondents by Professional Profile

Within the professional structure of the sample, general practitioners predominated (37/156, 23.7%), followed by surgeons (17/156, 10.9%), otorhinolaryngologists (12/156, 7.7%), internists (11/156, 7.1%), oncologists (10/156, 6.4%), and psychiatrists (9/156, 5.8%). The remaining medical specialties were represented in smaller numbers and were grouped into the category “other medical specialties,” which made it possible to simplify the presentation of the professional composition of the sample without losing its overall structure. The detailed distribution is presented in Table 2.

**Table 2.** Distribution of respondents by specialty groups (n=156).

Number	Specialty group	Results, n (%)
1	General practitioners	37 (23.7)
2	Surgeons	17 (10.9)
3	Otorhinolaryngologists (ENT^a^ specialists)	12 (7.7)
4	Internists	11 (7.1)
5	Oncologists	10 (6.4)
6	Psychiatrists	9 (5.8)
7	Clinical pharmacologists	8 (5.1)
8	Pediatricians	6 (3.8)
9	Urologists	5 (3.2)
10	Other medical specialties	41 (26.3)

a ENT: ear, nose, throat.

### Awareness, Telemedicine Formats, and AI (Q7-Q9)

The level of familiarity with telemedicine among physicians was heterogeneous. In routine practice, the most commonly used formats were telephone consultations (78/156, 50%), video consultations (69/156, 44.2%), chats and messaging applications (57/156, 36.5%), and mobile apps (48/156, 30.8%). Use of the KNTN was reported by 14.7% (23/156) of respondents, whereas use of wearable devices was reported by 5.8% (9/156). Telemedicine technologies incorporating AI elements were used regularly by 13.5% (21/156) of physicians and occasionally by 32.1% (50/156) of physicians, whereas 50.6% (79/156) reported not using them in their practice. Detailed data are presented in Table 3.

**Table 3.** Familiarity with telemedicine (Q7), telemedicine formats used (Q8, multiple response), and experience with the use of artificial intelligence (Q9).

Question and response categories	Results, n (%)^a^
How familiar are you with telemedicine technologies? (Q7)	
1: Not familiar at all	23 (14.7)
2: Slightly familiar	22 (14.1)
3: Moderately familiar	39 (25)
4: Familiar	48 (30.8)
5: Very familiar	24 (15.4)
Which formats of telemedicine do you use in your practice? (Q8)	
Kazakhstan National Telemedicine Network	23 (14.7)
Video consultations	69 (44.2)
Telephone	78 (50)
Mobile apps	48 (30.8)
Chats and messaging applications	57 (36.5)
Wearable devices	9 (5.8)
Have you used telemedicine technologies with artificial intelligence in your practice? (Q9)	
Yes, regularly	21 (13.5)
Yes, occasionally	50 (32.1)
No	79 (50.6)
Do not know/Not sure	6 (3.8)

a Percentages were calculated from valid n, and totals may not equal 100% due to rounding.

### Assessment of Usability, Effectiveness, and Technical Aspects

The distribution of responses to the scaled items showed a predominance of neutral and moderately positive ratings. The simplicity and intuitiveness of telemedicine platforms (Q10) were rated positively by 48.7% (76/156) of respondents, whereas 39.1% (61/156) selected a neutral response. The highest proportion of positive responses was observed for the item on the timeliness of patient condition monitoring (Q12), with 56.4% (88/156) of respondents providing a positive rating. The potential usefulness of telemonitoring for earlier detection of deterioration in a patient’s condition (Q13) was rated as fairly high or very high by 48.7% (76/156) of physicians, while an additional 41% (64/156) indicated moderate usefulness. This finding should be interpreted with caution, as it may reflect not only respondents’ practical experience but also their broader professional perceptions or expectations regarding telemonitoring. In relation to patients with chronic diseases (Q14), positive ratings accounted for 51.9% (81/256), whereas the perceived reduction in the need for face-to-face visits (Q15) was viewed more cautiously: Positive responses accounted for 41.1% (64/156), neutral responses accounted for 40.4% (63/156), and negative responses accounted for 18.6% (29/156). Technical stability remained the most vulnerable aspect, with only 35.9% (56/156) of participants giving a positive assessment of connection reliability and stability (Q17). Detailed data are provided in Table 4.

**Table 4.** Assessment of usability, clinical effectiveness, and connection stability (Q10, Q12-Q15, Q17).

Question and response categories	Results, n (%)^a^
Telemedicine platforms are simple and intuitive to use (Q10)	
Strongly disagree	8 (5.1)
Disagree	11 (7.1)
Neutral	61 (39.1)
Agree	62 (39.7)
Strongly agree	14 (9)
Telemedicine helps to monitor patient condition in a timely and effective manner (Q12)	
Strongly disagree	11 (7.1)
Disagree	8 (5.1)
Neutral	49 (31.4)
Agree	68 (43.6)
Strongly agree	20 (12.8)
Telemonitoring (if applied) allows earlier detection of patient deterioration (Q13)	
Not effective at all	6 (3.8)
Slightly effective	10 (6.4)
Moderately effective	64 (41)
Fairly effective	54 (34.6)
Very effective	22 (14.1)
Telemedicine is effective in the treatment of patients with chronic diseases (Q14)	
Not effective at all	9 (5.8)
Slightly effective	10 (6.4)
Moderately effective	56 (35.9)
Fairly effective	59 (37.8)
Very effective	22 (14.1)
Remote formats reduced the need for in-person visits to medical facilities (Q15)	
Not effective at all	13 (8.3)
Slightly effective	16 (10.3)
Moderately effective	63 (40.4)
Fairly effective	43 (27.6)
Very effective	21 (13.5)
The connection when using telemedicine platforms is stable and reliable (Q17)	
Strongly disagree	11 (7.1)
Disagree	24 (15.4)
Neutral	65 (41.7)
Agree	46 (29.5)
Strongly agree	10 (6.4)

a The sum of shares for each question equals 100% (minor deviations of ±0.1% are possible due to rounding).

### Readiness for Telemonitoring and Remote Care Formats

Within the section devoted to communication and telemonitoring, neutral or moderately positive attitudes predominated. The greatest uncertainty was observed for the item related to the accuracy of data transmission from wearable devices (Q18), where 57.1% (89/156) of respondents selected a neutral response option. Neutral or moderate responses were interpreted cautiously as they could potentially reflect ambiguity in perception, limited direct experience, absence of a firm opinion, or difficulty with evaluating the relevant aspect. At the same time, the effectiveness of establishing contact with patients through remote channels (Q19) and the preservation of communication quality (Q21) were rated more favorably, with positive responses outnumbering negative ones. Readiness to recommend telemonitoring at the national level was more often rated as moderate or high: 50% (78/156) of respondents indicated a moderate level of readiness, 26.9% (42/156) indicated a high level, and 9% (14/156) indicated a very high level. Detailed data are presented in Table 5.

**Table 5.** Communication and readiness for telemonitoring scale-up (Q18-Q21, Q23).

Question and response categories	Results, n (%)^a^
Data transmission from wearable devices (telemonitoring) works correctly and without errors (Q18)	
Strongly disagree	12 (7.7)
Disagree	13 (8.3)
Neutral	89 (57.1)
Agree	36 (23.1)
Strongly agree	6 (3.8)
I can effectively establish contact with the patient through telemedicine channels (Q19)	
Strongly disagree	8 (5.1)
Disagree	18 (11.5)
Neutral	63 (40.4)
Agree	54 (34.6)
Strongly agree	13 (8.3)
Patients feel comfortable during remote communication (Q20)	
Strongly disagree	9 (5.8)
Disagree	15 (9.6)
Neutral	74 (47.4)
Agree	48 (30.8)
Strongly agree	10 (6.4)
The remote format does not worsen the quality of my communication with the patient (Q21)	
Strongly disagree	8 (5.1)
Disagree	12 (7.7)
Neutral	67 (42.9)
Agree	58 (37.2)
Strongly agree	11 (7.1)
How ready are you to recommend the implementation of telemonitoring at the national level? (Q23)	
Very low readiness	11 (7.1)
Low readiness	11 (7.1)
Moderate readiness	78 (50)
High readiness	42 (26.9)
Very high readiness	14 (9)

a The sum of shares for each question equals 100% (minor deviations of ±0.1% are possible due to rounding).

### Organizational Aspects

The organizational aspects of telemedicine were characterized by a pronounced need for training and heterogeneity in regulatory and organizational support. Most respondents (110/156, 70.5%) indicated a need for additional training for the effective use of telemedicine platforms, while 76.3% (119/156) recommended the use of telemedicine primarily for select categories of patients. The development potential of telemedicine was more often rated as moderate or high (111/156, 71.2%).

At the same time, only one-third (52/156, 33.3%) of participants reported having approved protocols in place at their institutions, and the most common response regarding the distribution of responsibility was the absence of a specifically designated person (57/156, 36.5%). Among the priorities for further telemedicine development, the most frequently mentioned were internet connection quality (56/156, 35.9%) and the need for additional training (26/56, 16.6%). Detailed data are presented in Table 6.

**Table 6.** Organizational aspects including training needs, regulatory support, coordination, and telemedicine development potential (Q11, Q16, Q22, Q24-Q27).

Questions and response categories	Results, n (%)^a^
Do you need additional training for the effective use of telemedicine platforms? (Q11)	
Yes	110 (70.5)
No	29 (18.6)
Undecided	17 (10.9)
Would you recommend the use of telemedicine for specific patient categories (eg, chronic, rural)? (Q16)	
Yes	119 (76.3)
No	11 (7.1)
Undecided	26 (16.7)
How do you assess the potential for telemedicine development in your region/country? (Q22)	
Very low	8 (5.1)
Low	25 (16)
Moderate	80 (51.3)
High	31 (19.9)
Very high	12 (7.7)
Are there approved protocols or instructions for telemedicine services in your institution? (Q24)	
Yes	52 (33.3)
No	56 (35.9)
Undecided	48 (30.8)
Who is responsible for the organization and quality of telemedicine consultations in your institution? (Q25)	
Attending physician	17 (10.9)
Department leadership	33 (21.2)
Designated coordinator	49 (31.4)
No one specifically assigned	57 (36.5)
Which topics in telemedicine would you like to study to improve your qualifications? (Q26)	
Training/professional development (including skills, basic telemedicine, information, education)	99 (63.5)
Other	10 (6.4)
Do not know/No/Undecided	47 (30.1)
In your opinion, what should be improved for the development of telemedicine and telemonitoring in Kazakhstan? (Q27)	
Internet, connectivity	56 (35.9)
Education/professional development	26 (16.6)
Governance and funding issues	4 (2.6)
Technologies	4 (2.6)
Other	30 (19.2)
Do not know/No/Undecided	36 (23)

a The sum of shares for each question equals 100% (minor deviations of ±0.1% are possible due to rounding).

### Correlation Analysis

Spearman rank correlation analysis was conducted to assess the relationship between the level of familiarity with telemedicine (Q7) and perceptions of its usability and usefulness. Statistically significant positive correlations were identified between Q7 and Q10 (ρ=0.519; *P*<.001), Q12 (ρ=0.436; *P*<.001), Q13 (ρ=0.413; *P*<.001), Q14 (ρ=0.335; *P*<.001), and Q15 (ρ=0.389; *P*<.001). The strongest association was observed between familiarity with telemedicine and the perceived ease of use of telemedicine platforms (Q10), whereas associations with indicators of perceived clinical usefulness and organizational value were positive but less pronounced. Thus, a higher level of familiarity with telemedicine was associated with more positive evaluations of its usability and perceived effectiveness.

### Additional Stratified Analysis

The additional stratified analysis showed that ratings of telemedicine platform usability (Q10) differed significantly according to sex and years of work experience. Men gave higher ratings for platform usability than women (*P*=.006; adjusted *P*=.047 after correction for multiple comparisons). Significant differences were also identified by years of work experience (*P*=.005; adjusted *P*=.047), with the highest usability ratings observed among physicians with more than 20 years of experience compared with those in the group with 3 years to 5 years of experience. In addition, readiness to recommend the implementation of telemonitoring at the national level (Q23) was higher among men than among women (*P*=.001; adjusted *P*=.04).

Differences across age groups and workplace categories showed certain trends in some cases; however, in most instances, they did not remain statistically significant after correction for multiple testing. No consistent differences were identified between the compared groups in the evaluation of telemedicine effectiveness in the management of patients with chronic diseases (Q14).

Overall, the results indicate a heterogeneous perception of telemedicine among physicians in Kazakhstan: Despite moderately positive ratings of its usefulness and a statistically significant association between familiarity with telemedicine and physicians’ perceptions of it, substantial technical and organizational barriers remain, including insufficient connection stability, concerns about the accuracy of data transmission, and a high need for additional training. These findings point to a gap between the generally favorable perception of telemedicine and the persistent limitations affecting its practical implementation, which are discussed further in the Discussion section.

## Discussion

### Main Findings

This study showed that physicians in Kazakhstan had a generally moderately positive but heterogeneous perception of telemedicine. A substantial proportion of respondents gave positive assessments of its perceived clinical usefulness, particularly in the context of patient monitoring and the management of chronic diseases. At the same time, pronounced technical and organizational barriers remained, including connection instability, concerns about the accuracy of data transmission, the absence of local protocols, and a high need for additional training. Taken together, these findings suggest that a certain degree of readiness to adopt telemedicine solutions already exists within the professional community; however, their broader implementation continues to be constrained by infrastructural, organizational, and educational conditions.

### Interpretation of the Association Between Familiarity and Perception

The correlation analysis revealed a statistically significant positive association between physicians’ level of familiarity with telemedicine and their ratings of its usability, as well as its perceived clinical and practical usefulness. The strongest association was observed between familiarity with telemedicine and perceptions of the ease of use of telemedicine platforms (Q7-Q10; ρ=0.519), whereas associations with other aspects of telemedicine practice were also positive but less pronounced (Q7-Q12/15; ρ=0.335‐0.436). This suggests that a higher level of familiarity with telemedicine was associated with a more favorable perception of digital solutions.

At the same time, the findings do not permit causal inference. Greater familiarity may facilitate technology acceptance; however, the reverse scenario is also possible, whereby a more positive initial attitude increases physicians’ willingness to engage more deeply in digital practice. In addition, both variables may be influenced by external factors, including the level of technical infrastructure, organizational support, and the availability of training. The relatively stronger association between familiarity with telemedicine and perceptions of its ease of use may partly reflect the effect of prior experience or informational exposure, which should also be taken into account. Physicians who are already familiar with digital platforms may evaluate them more favorably not only because of the objective characteristics of the system but also because of greater confidence with using such solutions.

It should also be noted that, in this study, responses to items related to telemonitoring and wearable devices reflect, to some extent, physicians’ professional perceptions and expectations rather than their direct practical experience exclusively. Accordingly, these findings should be regarded as a preliminary indicator of attitudes toward these technologies rather than as direct evidence of their clinical effectiveness or technical reliability in real-world practice.

### Sociodemographic Differences

The additional stratified analysis showed that statistically significant differences in evaluations of telemedicine platforms were not observed across all sociodemographic characteristics. The most consistent differences were identified by sex and years of work experience with regard to platform usability and by sex with regard to readiness to recommend the implementation of telemonitoring at the national level. In particular, men gave higher ratings for the usability of telemedicine platforms and were more likely to support their broader implementation. In addition, higher usability ratings were observed among physicians with more than 20 years of work experience compared with those with 3 years to 5 years of experience. In contrast, differences across age groups and workplace categories did not remain statistically significant in most cases after correction for multiple testing. Collectively, these findings do not support the simplified assumption that attitudes toward telemedicine are determined primarily by age or length of professional experience; rather, they point to a more complex set of factors shaping perceptions of digital solutions.

### Comparison With the International Literature

Overall, the findings of this study are consistent with international publications identifying technical limitations, insufficient digital preparedness of health care staff, a lack of standardized protocols, and limited institutional support as major barriers to telemedicine implementation [5,23-26]. The high need for additional training identified in our sample is also consistent with published evidence showing that training is regarded as one of the key prerequisites for the successful implementation of digital health services [27-29]. Thus, this study does not merely reproduce international findings; rather, it demonstrates that similar systemic barriers are also evident in the context of Kazakhstan.

Particular attention should be paid to issues of safety, confidentiality, and trust in digital health solutions. In this study, concerns about the accuracy of data transmission and the instability of digital channels may be regarded as factors that potentially limit physicians’ trust in telemedicine services. These observations are consistent with international evidence showing that trust in telemedicine is closely linked to infrastructure reliability, transparency in data handling, and adherence to confidentiality principles [30]. This underscores that the technical reliability of telemedicine solutions has not only organizational but also ethical and regulatory significance.

The findings also make it possible to identify conditions under which telemedicine implementation may be more sustainable. These include organizational support, the presence of local protocols, staff training, and more active physician engagement in digital processes. These conclusions are consistent with international publications in which administrative support, procedural standardization, and systematic staff training are regarded as key conditions for the sustainable implementation of telemedicine solutions [27,29,31]. In addition, the results of this study highlight the importance of context-specific adaptation of digital solutions to the conditions of a particular health care system, the level of infrastructural readiness, and the characteristics of clinical practice, which is also consistent with international evidence [31-33].

### Practical and Policy Implications

From a practical perspective, the findings make it possible to identify several priority areas for the health care system of Kazakhstan. First, systematic educational programs for physicians appear to be necessary, aimed not only at mastering digital platforms but also at building confidence in their clinical and organizational use. Second, telemedicine processes require standardization, including the development of local protocols, referral pathways, and a clearer distribution of responsibility for telemedicine consultations. Third, without strengthening the technical infrastructure—above all, the quality of internet connectivity, the stability of digital channels, and the reliability of data transmission—even a moderately positive attitude among physicians is unlikely to translate into sustained telemedicine use. Accordingly, telemedicine expansion should be accompanied by coordinated measures at both institutional and health-system levels, including workforce training, local protocol development, clearer accountability structures, and minimum technical readiness standards.

More broadly, the results are relevant not only to the practice of individual health care organizations but also to the discussion of managerial and policy-organizational approaches to the digital transformation of health care. The observed differences in levels of digital awareness and perceptions of telemedicine highlight the need for targeted measures to strengthen physicians’ digital competencies, reduce disparities in physicians’ digital readiness across professional and organizational contexts, and support the phased implementation of telemedicine solutions in line with the actual readiness of organizations. The findings may also serve as a reference point for improving continuing professional development programs, developing more structured organizational mechanisms for telemedicine implementation, and further advancing national approaches to health care digitalization in Kazakhstan. Thus, despite the pilot nature of the study, its results may be regarded as a preliminary empirical basis for future larger-scale research and for discussion of institutionally supported approaches to telemedicine implementation [27-29,31].

### Strengths, Limitations, and Future Research Directions

The strengths of this study include the use of the previously developed and validated TUQ-MAST-KZ instrument, adapted to the national context of Kazakhstan, as well as the inclusion of physicians working in different types of health care organizations and across diverse care settings. At the same time, the study has several limitations. First, the data were based on participant self-report and may therefore be subject to reporting bias and subjective interpretation. Second, a convenience nonprobability sample was used, with questionnaire dissemination through professional networks, creating a risk of self-selection bias and preventing the calculation of an exact response rate. Therefore, the findings should be regarded as exploratory and potentially somewhat optimistic estimates of physicians’ perceptions rather than as representative national estimates. In particular, physicians with a stronger prior interest in telemedicine and digital technologies may have been more likely to participate, which could potentially have resulted in more favorable assessments of telemedicine solutions compared with the overall physician population. At the same time, physicians with lower levels of digital engagement, those not connected to professional online networks, those less likely to participate in online surveys, or those less interested in telemedicine may have been underrepresented in the sample. Accordingly, the findings should be interpreted with caution and cannot be considered nationally representative of physicians in Kazakhstan.

Third, the study had a cross-sectional design, and the observed associations therefore cannot be interpreted as causal. Fourth, objective technical parameters of the digital infrastructure were not assessed. Finally, although information on participants’ professional profiles was available, no separate statistical analysis of differences across medical specialties was conducted because of the high fragmentation of the sample, the small size of a number of subgroups, and the limited interpretability of such comparisons within the framework of a pilot study. An additional limitation is that the items related to telemonitoring and wearable devices may have reflected not only user experience but also respondents’ professional perceptions, expectations, and general attitudes toward these technologies.

Promising directions for future work include studies with larger and more structured samples of physicians to examine differences across professional and regional groups, as well as interventional studies of educational programs assessing how training influences perceptions of telemedicine and readiness to use it. Further investigation is also needed for pilot telemonitoring projects, including assessments of data quality, organizational feasibility, and economic efficiency, as well as the development and testing of standard clinical and organizational protocols for integrating telemedicine solutions into the practice of health care organizations in Kazakhstan. The international literature also shows that barriers to telemedicine implementation may differ substantially by clinical specialty. For example, in pediatrics, issues of confidentiality and parental trust are of particular importance [9,34]; in psychiatry, interdisciplinary collaboration and overcoming professional resistance are especially relevant [27,32]; and in telerehabilitation, key concerns include technical limitations, organizational coordination, and doubts about the accuracy of remote patient assessment [33,35,36]. Because specialty-specific differences were not specifically analyzed in this study, this issue requires separate investigation in larger and professionally stratified samples.

### Conclusion

This study made it possible to identify the key barriers and factors influencing perceptions of telemedicine among physicians in Kazakhstan. Using the TUQ-MAST-KZ instrument, the study provided an initial assessment of physicians’ familiarity with digital technologies, their perceptions of usability, and the barriers limiting the implementation of telemedicine in clinical practice. The findings may serve as a reference point for the development of targeted training programs, the standardization of protocols, the improvement of infrastructure, and the strengthening of organizational support. Thus, TUQ-MAST-KZ represents a promising and context-adapted instrument for assessing perceptions of telemedicine and may be used in subsequent larger-scale studies, as well as in discussions of approaches to the phased implementation of telemedicine solutions in the Republic of Kazakhstan.

## Supplementary material

10.2196/83667 Multimedia Appendix 1 Ethical approval.

10.2196/83667 Multimedia Appendix 2 Telehealth Usability Questionnaire – Model for Assessment of Telemedicine, Kazakhstan Version.

## Abbreviations

AI: artificial intelligence
KNTN: Kazakhstan National Telemedicine Network
MAST: Model for Assessment of Telemedicine
TUQ: Telehealth Usability Questionnaire
TUQ-MAST-KZ: Telehealth Usability Questionnaire-Model for Assessment of Telemedicine-Kazakhstan
