# Supplementary material for: Assessment of Telemedicine Perceptions, Usability, and Implementation Barriers Among Physicians in Kazakhstan Using the Telehealth Usability Questionnaire-Model for Assessment of Telemedicine-Kazakhstan Version (TUQ-MAST-KZ) Questionnaire: Pilot Cross-Sectional Survey Study
Source: JMIR Form Res. 2026 Apr 7;10:e83667. doi: 10.2196/83667 (PMC13055950; doi:10.2196/83667)
Supplement: Multimedia Appendix 1 [file formative-v10-e83667-s001.pdf]

ОДОБРЕНО ЛКпБ
АО «ЮКМА»

ТҮПНҰСҚА

Date of approval: April 25, 2025
Approval number: №3

## Approval letter

This is to certify that an ethics approval was granted to Kulzhamila Kenessova, PhD student at JSC "South Kazakhstan Medical Academy", for the study titled:

"Digital healthcare: an evaluation of the effectiveness of telemedicine and remote medical services"

The proposal was reviewed by the Local Bioethics Commission of JSC "South Kazakhstan Medical Academy" and received confirmation that it does not violate bioethical norms and standards.

Approval was issued for academic and publication purposes.

Domestic scientific consultants:Assoc. Prof. Marya Anartayeva, MD, DMedSc

PhD Kanatzhan Kemelbekov

Foreign scientific consultant:Assoc. Prof. Olena Zymba, PhD

Chairman of the Commission,
Assoc. Prof. ________________ Zh.A. Kauyzbay

Secretary of the Commission: ________________ D.M.Batyrbek
